# Supplementary figures and images for: Polyphenol-rich Trapa quadrispinosa pericarp extract ameliorates high-fat diet induced non-alcoholic fatty liver disease by regulating lipid metabolism and insulin resistance in mice
Source: PeerJ. 2019 Nov 29;7:e8165. doi: 10.7717/peerj.8165 (PMC6886490; doi:10.7717/peerj.8165)

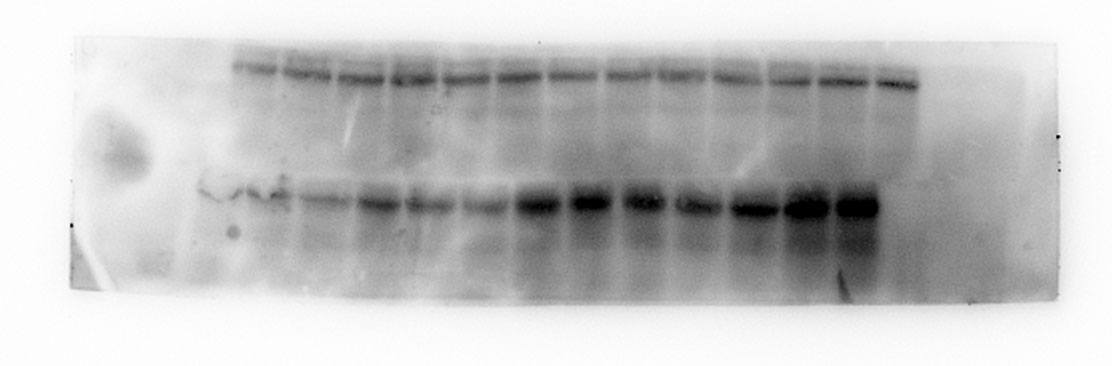

Supplement: Supplemental Information 2 — Full-length uncropped blots for Figs. 6A–6E. [file peerj-07-8165-s002.zip › raw-data-wb/Fig 6A-AMPK.jpg]

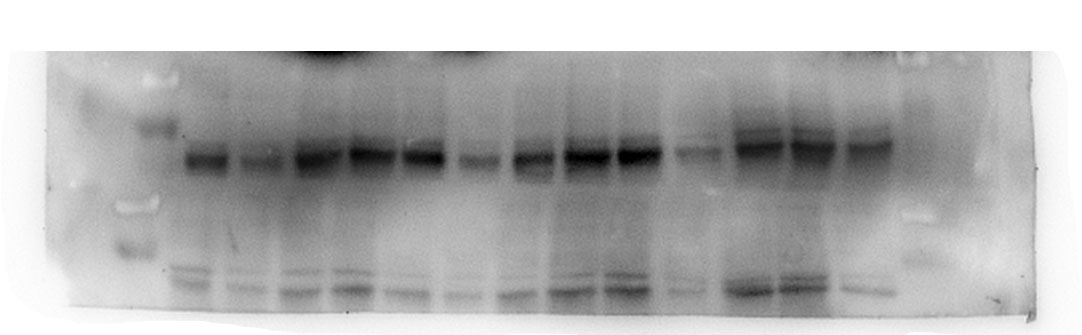

Supplement: Supplemental Information 2 — Full-length uncropped blots for Figs. 6A–6E. [file peerj-07-8165-s002.zip › raw-data-wb/Fig 6A-p-AMPK.jpg]

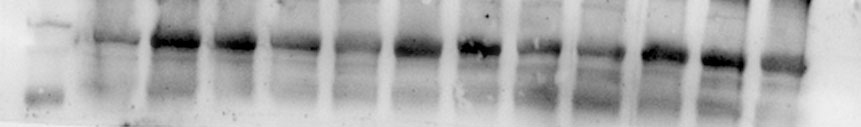

Supplement: Supplemental Information 2 — Full-length uncropped blots for Figs. 6A–6E. [file peerj-07-8165-s002.zip › raw-data-wb/Fig 6B-SREBP.jpg]

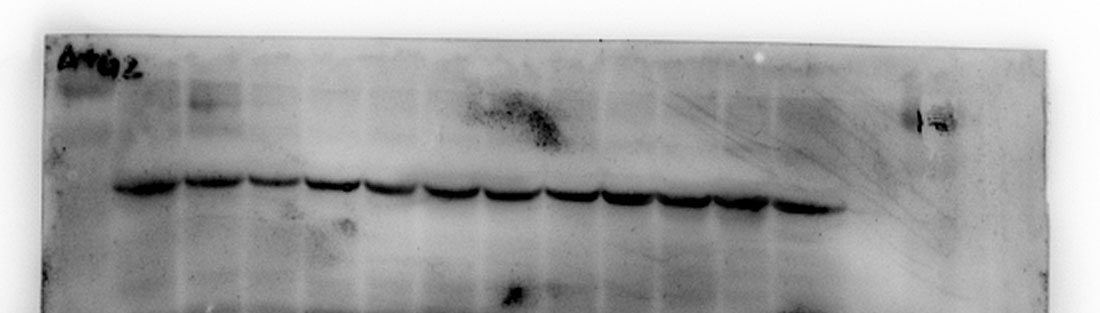

Supplement: Supplemental Information 2 — Full-length uncropped blots for Figs. 6A–6E. [file peerj-07-8165-s002.zip › raw-data-wb/Fig 6B-b-ACTIN.jpg]

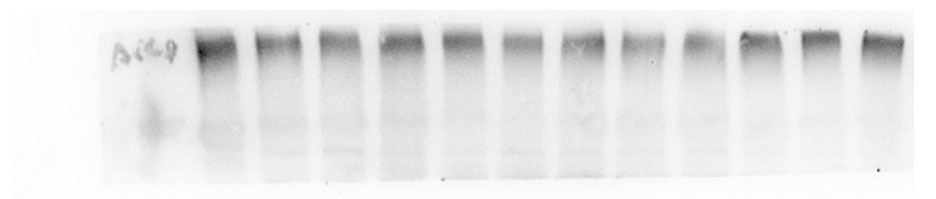

Supplement: Supplemental Information 2 — Full-length uncropped blots for Figs. 6A–6E. [file peerj-07-8165-s002.zip › raw-data-wb/Fig 6C-ACC.jpg]

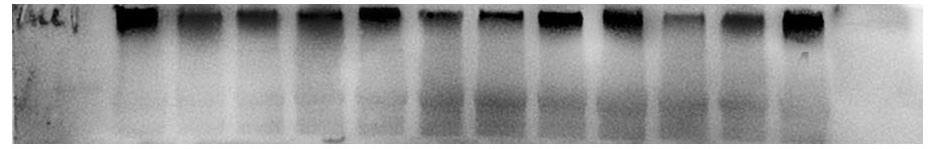

Supplement: Supplemental Information 2 — Full-length uncropped blots for Figs. 6A–6E. [file peerj-07-8165-s002.zip › raw-data-wb/Fig 6C-p-ACC.jpg]

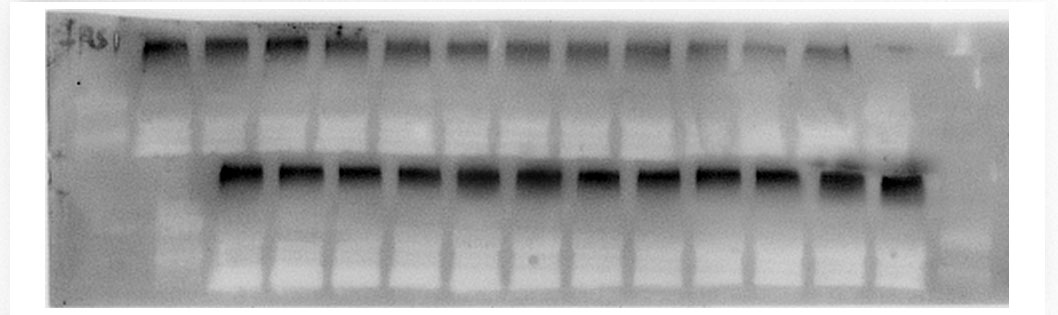

Supplement: Supplemental Information 2 — Full-length uncropped blots for Figs. 6A–6E. [file peerj-07-8165-s002.zip › raw-data-wb/Fig 6D-IRS.jpg]

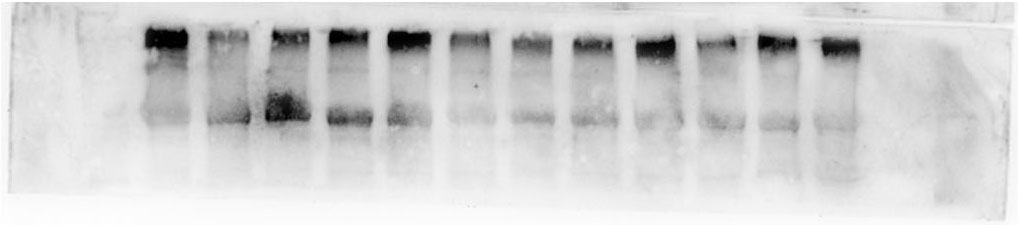

Supplement: Supplemental Information 2 — Full-length uncropped blots for Figs. 6A–6E. [file peerj-07-8165-s002.zip › raw-data-wb/Fig 6D-p-IRS.jpg]

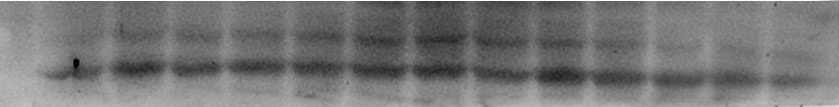

Supplement: Supplemental Information 2 — Full-length uncropped blots for Figs. 6A–6E. [file peerj-07-8165-s002.zip › raw-data-wb/Fig 6E-AKT.jpg]

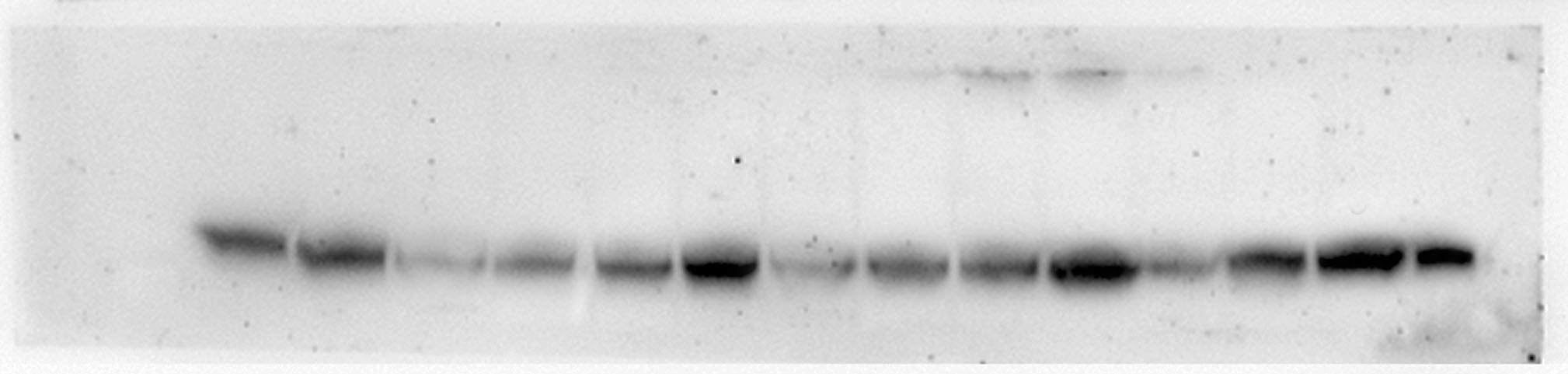

Supplement: Supplemental Information 2 — Full-length uncropped blots for Figs. 6A–6E. [file peerj-07-8165-s002.zip › raw-data-wb/Fig 6E-p-AKT.jpg]
